# Formation Energy Prediction and Feature Analysis of Lead-Free Double Perovskites Based on Deep Learning

**DOI:** 10.3390/molecules31183175

**Published:** 2026-09-10

**Authors:** Beibei Wang, Juan Wang

**Affiliations:** 1 School of Electronic Information, Xijing University, Xi’an 710123, China; wangbeibei@xijing.edu.cn; 2 Xi’an Key Laboratory of Advanced Photo-Electronics Materials and Energy Conversion Device, School of Materials and New Energy, Xijing University, Xi’an 710123, China

**Keywords:** lead-free double perovskite, formation energy, thermodynamic stability, deep learning, SHAP interpretability

## Abstract

Lead-free double perovskites (LFDPs) are emerging as promising low-toxicity and thermally stable candidates to replace lead-based perovskites in photovoltaic and optoelectronic devices. However, the rational design of high-stability LFDPs is severely constrained by the low efficiency of conventional experiments and density functional theory (DFT) calculations, as well as the limited accuracy and interpretability of existing machine learning models. To address these limitations, this study employed classic deep learning models to predict the DFT-calculated formation energy of the A_2_BB’X_6_ lead-free double perovskite material. Based on a dataset of 1027 DFT-calculated samples, four deep learning models, namely MLP, deep ensemble, PINN, and Transformer, were constructed to accurately predict the thermodynamic stability of LFDPs using formation energy as the core evaluation index. Combined with correlation analysis and SHapley Additive exPlanations (SHAP) interpretable learning, the optimal geometric stability window for LFDPs was identified, with a tolerance factor of 0.8~0.9 and an octahedral factor of 0.4~0.8. Comparative model validation demonstrates that the MLP model exhibits the best predictive performance, achieving a mean absolute error (MAE) of 0.0951 and a coefficient of determination (R^2^) of 0.9147 on the test set. SHAP analysis further reveals that the electronegativities of B1 and B2 cations are the dominant electronic factors governing the formation energy and phase stability of LFDPs, with a positive synergistic effect, while lattice size parameters (e.g., B2 ionic radius and B1 van der Waals radius) act as secondary influencing factors. This work constructs a high-precision, physically interpretable data-driven regression benchmark for formation energy, delivers multi-dimensional mechanistic interpretation of feature contributions, and acts as a reference for high-throughput material screening.

## 1. Introduction

With the global energy system undergoing a shift toward clean and low-carbon development, the issues of energy shortages and environmental pollution are increasingly prominent. Developing efficient, green, and stable novel optoelectronic materials has become a core need to promote sustainable development in photovoltaics, photodetection, and related fields [1]. Perovskite materials exhibit outstanding advantages, including a direct and tunable bandgap, high absorption coefficient, excellent carrier mobility, and low fabrication cost. They have shown broad application prospects in solar cells, photodetectors, and light-emitting diodes [2,3].

III-V semiconductor nanowires are another high-performance optoelectronic candidate with remarkable light-harvesting capability and a tailorable spectral response. Yet their large-scale practical deployment is hindered by stringent fabrication prerequisites. High-quality epitaxial growth, precise multicomponent stoichiometry regulation, and delicate core–shell interface passivation all impose high technical and cost thresholds [4]. Systematic core–shell nanowire research further confirms that rational band alignment across heterointerfaces is the prerequisite for efficient separation and collection of photogenerated charge carriers [5]. Although machine learning (ML)-driven optimization has been advanced for III-V nanowire heterostructures, costly epitaxy still limits broad material screening [6]. Compared with III-V nanowires, perovskite systems offer complementary merits: flexible compositional bandgap engineering, low temperature, solution processable manufacturing routes, and superior light absorption coefficients, without requiring ultrahigh-vacuum epitaxy. Still, toxic lead constituents and insufficient operational thermal/humidity stability remain critical bottlenecks restricting their commercial rollout, driving the urgent exploration of LFDP substitutes as promising environmentally friendly alternatives [7].

Compared with traditional silicon-based materials, perovskites offer significantly reduced production costs, better environmental friendliness, and rapidly improving power conversion efficiencies, which render them competitive candidate materials for next-generation photovoltaic technologies [8,9]. However, the toxicity of lead and the poor thermal stability of mainstream lead-based perovskites limit their industrialization and pose ecological safety risks [10,11]. Therefore, developing lead-free, high-stability perovskite alternatives has become an urgent research priority.

LFPs include single and double perovskites. Although both categories can serve as lead substitutes, they exhibit substantial performance differences. Lead-free single perovskites replace Pb^2+^ with a single metal cation; while straightforward to fabricate, they are prone to structural distortion and high defect densities, which limit their structural stability and the tunability of optoelectronic properties, rendering them frequently incapable of satisfying practical application requirements [12]. In contrast, LFDPs, with dual coordinated metal cations, deliver more prominent performance advantages and are the most promising industrial alternatives to lead-based perovskites [13]. In this type of perovskite material, Pb^2+^ is substituted by non-toxic or low-toxicity mixed metal cations (e.g., Sn^2+^, Ge^2+^, Bi^3+^). The synergistic effect between two B-site cations optimizes crystal symmetry and bonding; double perovskites reduce defect densities and significantly enhance stability. Moreover, the rich choice of A, B1, B2, and X-site elements yields a vast compositional design space to flexibly tune bandgaps, carrier mobilities, and other key parameters for different optoelectronic applications. However, the enormous compositional and structural space leads to an exponentially growing number of candidates. Several prior high-throughput DFT efforts have attempted to explore A_2_BB’X_6_ double-perovskite chemical spaces, yet traditional experimental characterization and DFT calculations are still constrained by long testing and calculation cycles, high economic costs and limited research coverage, which hinders the efficient screening of high-performance candidate materials [14,15]. Stability under service conditions (high temperature, high humidity, illumination) remains a challenge; some materials are metastable even at room temperature and undergo accelerated phase transitions, which severely restrict practical applications [16]. Consequently, efficiently identifying LFDPs with high stability and excellent optoelectronic properties from a vast chemical space is a key scientific challenge in perovskite materials research.

Formation energy is a critical indicator for evaluating the synthetic feasibility, structural stability, and application potential of LFDPs [17]. It directly reflects the difficulty of synthesis at thermodynamic equilibrium; lower formation energies imply easier synthesis experimentally. In recent years, machine learning has been successfully applied to predict perovskite formation energies, providing a new pathway for materials screening. For example, Liang et al. used Extreme Gradient Boosting (XGBoost) on a dataset of 469 compounds to predict 22 new stable perovskites, achieving an R^2^ of 0.959 [18]. Bartel et al. developed a one-dimensional explicit tolerance factor τ via the Sure Independence Screening and Sparsifying Operator (SISSO) algorithm to predict perovskite structural stability, achieving 92% accuracy on 576 ABX_3_ perovskites; when extended to A_2_BB’X_6_ double perovskites, accuracy remained around 91% [19]. Chen et al. combined ML and DFT to systematically investigate structural stability and thermoelectric properties in halide perovskites, using neuroevolution potentials with molecular dynamics to discover temperature-driven phase transitions, demonstrating ML’s potential for finite-temperature dynamic stability prediction [20]. Despite these advances, only a small set of studies have targeted formation energy prediction specifically for A_2_BB’X_6-_type LFDPs; many existing models are trained on general perovskite datasets dominated by lead–halide compositions [21]. Challenges persist in applying ML to formation energy prediction of LFDPs: limited data quality and coverage, insufficient accuracy for thermodynamic criteria, the lack of embedded physical mechanisms, and poor interpretability, hindering identification of key stability factors [22,23]. These issues hinder effective guidance for designing stable LFDPs, highlighting the need for efficient, accurate, and interpretable methods to elucidate critical stability determinants and overcome current bottlenecks.

Deep learning (DL), with powerful nonlinear fitting and automatic feature extraction capabilities, is shifting materials performance prediction from inefficient trial-and-error and first-principles paradigms to efficient data-driven modes, showing great promise in perovskite research and development [24]. Different DL models offer distinct advantages. MLP can fit complex nonlinear relationships between composition and properties [25]; Ye achieved high-accuracy DFT formation energy prediction for C_3_A_2_D_3_O_12_ garnets and ABO_3_ perovskites using MLP with only two descriptors (Pauling electronegativity and ionic radius), with an MAE of 7–10 meV·atom^−1^ and 20–34 meV·atom^−1^, respectively [26]. Ensemble learning can achieve accurate predictions at low computational cost, shortening device design cycles and supporting industrialization of perovskite solar cells [27]. Fang’s comprehensive ensemble model accurately predicts formation energy, convex hull energy, and bandgap, with an MAE as low as 0.146 eV, 0.041 eV, and 0.396 eV, respectively, approaching first-principles accuracy [28]. PINNs incorporate physical priors into the model structure to address small-sample and mechanism-deficit issues that limit predictive accuracy [29]. Hammad’s ThermoLearn PINN integrates domain thermodynamic principles to predict inorganic thermodynamic properties accurately, addressing small datasets and out-of-distribution (OOD) prediction challenges faced by traditional and standard ML methods [30]. Transformers, via self-attention, capture long-range correlations and complex interatomic interactions in crystals, enhancing feature representation [31]. Jin’s CrystalTransformer, using CrystalTransformer-based universal atomic embeddings (CT-UAEs) and Transformer attention, improved perovskite formation energy prediction relative to MatErials Graph Network (MEGNET), reducing the MAE from 0.032 to 0.021 (34% improvement), mitigating data scarcity and improving generalization [32]. However, at present, there is a lack of dedicated DL prediction frameworks for the formation energy of LFDPs, as well as systematic interpretive analysis of the key structural and compositional features that govern material stability. Our prior LFDP optoelectronics work used identical DL workflows to predict A_2_BB’X_6_ bandgaps [33]. Yet bandgap predictions fail to assess perovskite synthetic feasibility and phase stability, critical for fabrication and device durability. Although several DL works have studied halide double perovskites, few extend to oxide, fluoride and chloride LFDP families. DL is thus underexploited for stability targeted multi-anion LFDP design and screening.

Traditional experiments and DFT calculations hinder the large-scale screening of LFDPs [34]. Although existing machine learning models can predict perovskite formation energy, they still present notable limitations. Most current studies rely on tree-based models, including XGBoost and random forests, without systematically comparing deep learning architectures such as MLP, deep ensemble, PINN, and Transformer under fixed data partitioning. Furthermore, existing models primarily focus on halide perovskites while neglecting oxide, fluoride, and chloride systems [35]. In addition, most works evaluate model performance only using R^2^ and MAE, lacking SHAP-based interpretability to reveal synergies between geometric and electronic descriptors; interpretability-driven material discovery for LFDPs remains rare in the literature. The absence of multi-dimensional screening criteria restricts mechanistic understanding and high-throughput material screening. To address these gaps, this work systematically benchmarks multiple models on 1027 DFT-calculated A_2_BB’X_6_ samples under a fixed 8:2 train–test split. With SHAP quantification, we identify the dominant electronic effect of B-site electronegativity and the secondary geometric effect of lattice parameters, as well as their positive synergy. Beyond conventional single-tolerance-factor criteria, we establish a novel geometric electronic dual-screening window. Balancing prediction accuracy and physical interpretability, the derived screening rules effectively guide LFDP material design and substantially reduce the trial-and-error costs of DFT computations and experimental synthesis.

**Figure 1:**
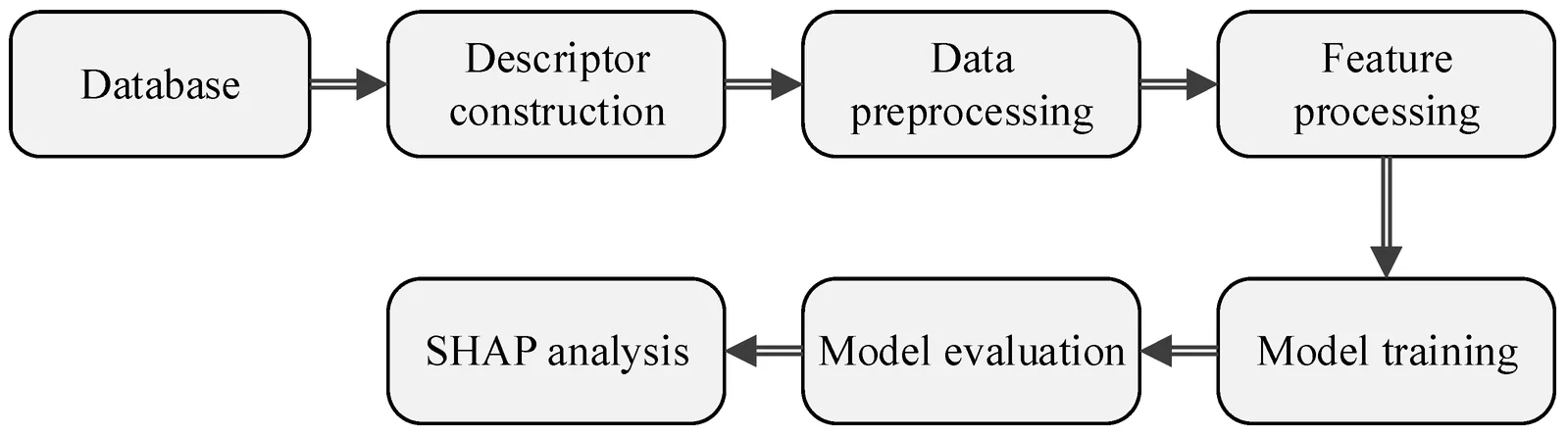
LFDP formation energy prediction and interpretability analysis: overall process.

The workflow implemented in this study is shown in Figure 1, including dataset construction, feature engineering, model training, and SHAP analysis. We constructed a formation energy prediction model for A_2_BB’X_6_ lead-free double perovskite materials and used SHAP analysis to explore the structure–performance relationship. This study provides a repeatable benchmark method that differs from previous studies: it focuses on formation energy, covers multiple anion systems, uses fixed data partitioning for a comprehensive comparison of multiple models, and proposes dual geometric–electronic feature selection rules through SHAP.

## 2. Results and Discussion

### 2.1. Feature Processing

For accurate characterization of compositional and structural properties, we built an LFDP descriptor system of 50 key physicochemical and structural features covering intrinsic atomic properties of the A, B1, B2, and X sites and crystal geometric parameters. Table 1 lists all descriptors alongside their categories, references, units, and detailed physical interpretations.

Data quality control is first implemented by removing outliers and samples with missing values. Next, MinMaxScaler is adopted to normalize features to the range of [0, 1] [36]. This approach effectively mitigates issues arising from disparate feature scales, improves the fairness of feature weighting, accelerates optimizer convergence, enhances training efficiency, and boosts model stability and predictive performance [37]. In addition, MinMaxScaler enables inverse mapping for the recovery of original feature values. The mathematical definition of MinMax normalization is expressed by Formula (1) [38]:

(1) $$X_{norm}=\frac{X-X_{min}}{X_{max}-X_{min}}$$

where *X* is the raw value, *X_min_* and *X_max_* are the minimum and maximum in the training set, and *X_norm_* is the normalized value. This alleviates scale imbalance, ensures fair feature weighting, accelerates convergence, and stabilizes DL training and predictions.

Boxplots and kernel density estimates (KDE) were adopted for data visualization [39]. Axis standardization was performed to eliminate unit discrepancies and enable the unified presentation of all raw features (Supplementary Figure S1). Boxplot results indicate limited outliers in structural features, including ionic radii and tolerance factor, with the majority of tolerance factors falling within the 0.7–1.0 stability range. Atomic and electronic features exhibit higher data dispersion and a greater number of outliers. Thermodynamic features cover broad value ranges and present obvious skewness as well as extreme outliers. Overall, no systematic batch errors are detected in the dataset, which guarantees high sample reliability. KDE analysis reveals that structural features follow approximately unimodal normal distributions; atomic mass and ionization energy present multimodal distributions, which reflect the data stratification of A, B1, B2 and X constituent elements. Energy-related features show right-skewed distributions with long tails, and several extremely high values may impede the fitting of conventional models. The dataset covers five categories of features, namely crystal structure, atomic electronics, thermodynamics, and stability-associated geometric factors. All features possess definite physical implications and strong discriminative capability, making the dataset applicable to the performance prediction and high-throughput screening of perovskite materials.

**Table 1:** Key descriptors for predicting the formation energy of LFDPs.

Parameter Category	Feature Names	Unit	Description	Ref.
Ionic structural radii	A/B1/B2/X_ionic_radius	Å	ionic radii at each site	Ref. [40]
	A/B1/B2/X_van_der_waals_radius	Å	van der Waals radius	
Electrochemical atomic features	A/B1/B2/X_electronegativity	None	electronegativities at four sites	Ref. [41]
	A/B1/B2/X_electron_affinity	eV	electron affinities	
	A/B1/B2/X_first_ionization energy	eV	first ionization energies	
	A/B1/B2/X_second_ionization energy	eV	second ionization energies	
	A/B1/B2/X_third_ionization energy	eV	third ionization energies	
Basic atomic intrinsic properties	A/B1/B2/X_atomic_mass	g/mol	atomic masses	Ref. [42]
	A/B1/B2/X_atomic_number	None	atomic numbers	
Thermophysical parameters	A/B1/B2/X_melting_point	K	elemental melting points	Ref. [43]
	A/B1/B2/X_boiling_point	K	boiling points	
	A/B1/B2/X_thermal_conductivity	W·m^−1^·K^−1^	elemental thermal conductivities	
Geometric stability factors	tolerance_factor, octahedral_factor	None	tolerance and octahedral factors	Ref. [44]

### 2.2. Feature Correlation Analysis

Feature selection is critical in ML workflows, aiming to identify an optimal subset of representative, task-relevant features from the high-dimensional set [45]. This reduces dimensionality, removes noise and redundancy, improves predictive accuracy and generalization, and enhances interpretability. In practice, Pearson correlation is often used to quantify linear relationships between features and target variables, retaining strongly correlated features [46]. The Pearson coefficient, a classic measure of linear correlation between continuous variables, is widely used in preliminary screening. Its mathematical expression is shown in Formula (2) [47].

(2) $$\rho_{X,Y}=\frac{\sum_{i=1}^{n}\left(X_{i}-X^{-})(Y_{i}-Y^{-}\right)}{\sqrt{\sum_{i=1}^{n}{\left(X_{i}-X^{-}\right)}^{2}\sum_{i=1}^{n}{\left(Y_{i}-Y^{-}\right)}^{2}}}$$

**Figure 2:**
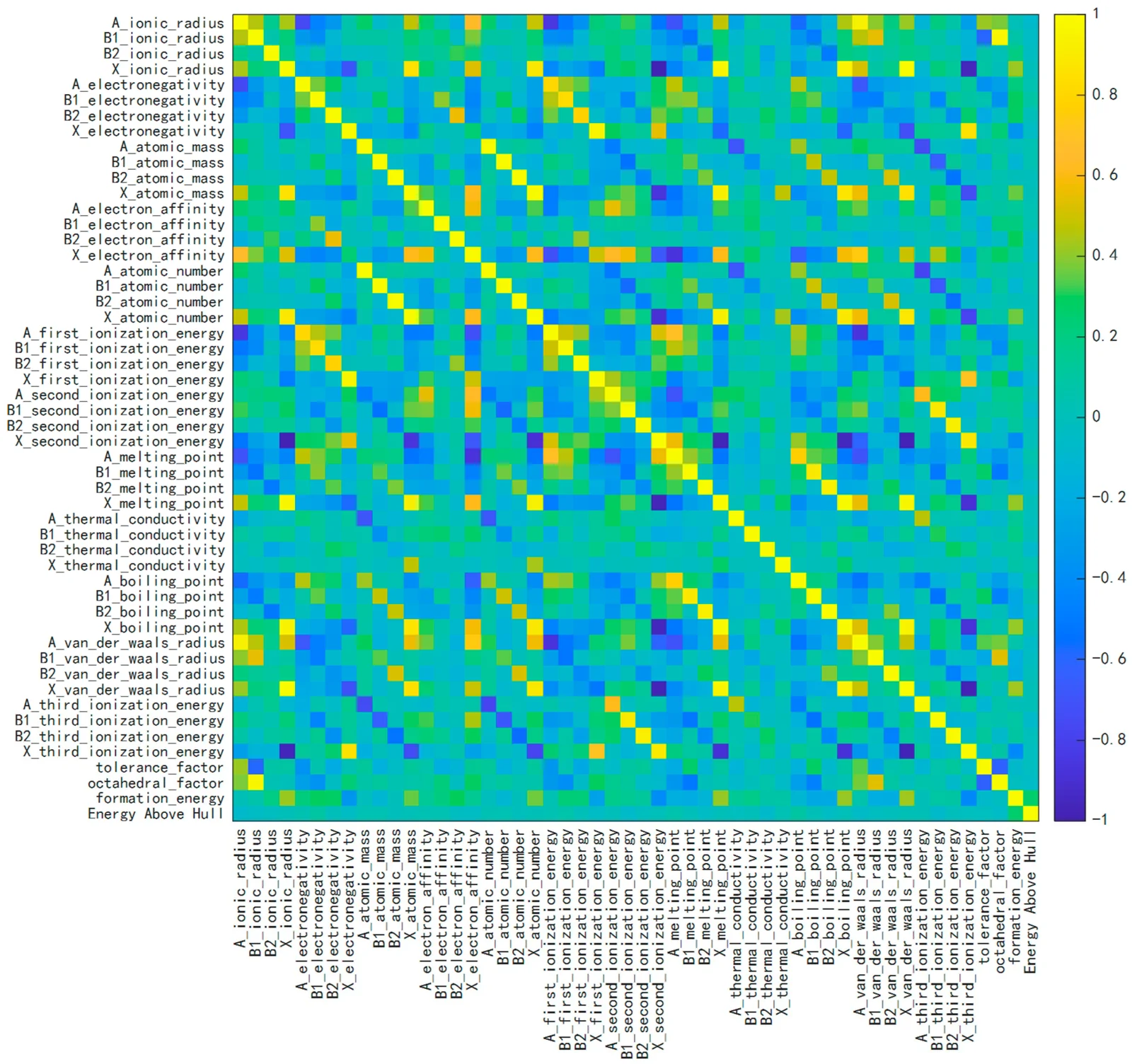
Heatmap of Pearson correlation coefficient for predictive features of double-perovskite formation energy.

Figure 2 shows a Pearson correlation heatmap between multi-source features and formation energy for LFDPs. Axes cover intrinsic physicochemical parameters at A, B1, B2, and X sites. The color bar maps coefficient magnitudes: bright yellow approaches 1 (strong positive correlation), dark blue approaches −1 (strong negative), and green near 0 (weak linear correlation). The main diagonal (yellow) represents self-correlation. The results indicate strong positive correlations among homologous elemental features across different sites, revealing pronounced multicollinearity and informing subsequent feature selection and redundancy removal. Overall, single basic elemental parameters show weak linear correlations with thermodynamic stability, while tolerance and octahedral factors exhibit moderate correlations, consistent with classical crystal chemistry heuristics. These findings suggest that double-perovskite phase stability arises from complex, multi-feature nonlinear couplings, beyond linear analysis, motivating the use of DL to discover higher-order, implicit relationships for high-throughput screening and rational design.

**Figure 3:**
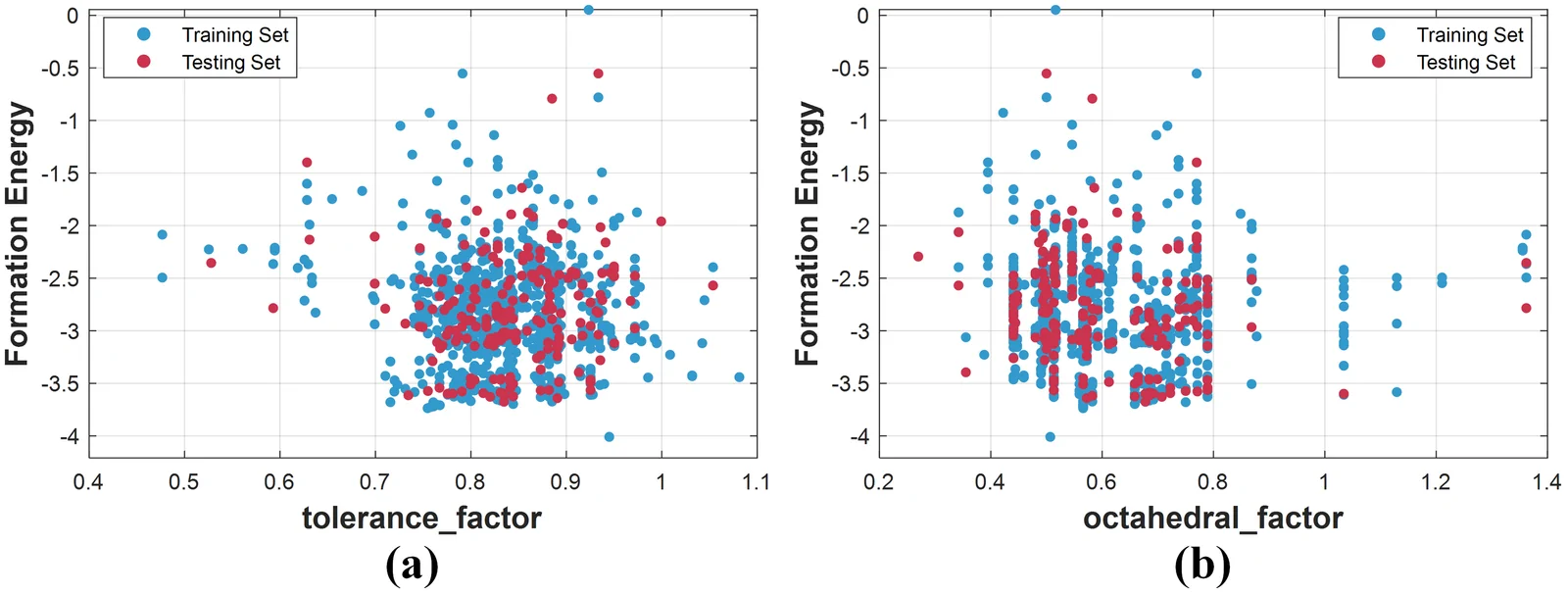
Visualization of sample distribution of geometric characteristics and formation energy for LFDP dataset. (**a**) Two-dimensional scatter plot of formation energy versus tolerance factor; (**b**) two-dimensional scatter plot of formation energy versus octahedral factor.

Figure 3a,b show 2D scatter plots of formation energy versus tolerance factor and octahedral factor, respectively. Blue and red points are training and test samples, respectively. Their overlapping distributions indicate consistent splits and good representativeness, supporting generalization. From a geometric perspective, compared with the octahedral factor, the tolerance factor has a stronger regulatory effect on the thermodynamic stability of the material. Although both represent the degree of crystal ion size matching, geometric mismatch introduces lattice distortion and strain energy, which affects thermodynamic stability. From the data correlation, it can be seen that the tolerance factor has a higher correlation with the formation energy. A large number of samples with tolerance factors in the range of 0.8~0.9 correspond to lower formation energies and better thermodynamic stability; once deviated from this range, the geometric compatibility of the crystal decreases, and the risk of thermodynamic instability increases. The tolerance window of the octahedral factor is 0.4~0.8. Within this range, the system is more likely to present a stable structure with a low formation energy. An excessively high octahedral factor will exacerbate the mismatch of the coordination environment and weaken the bond strength. In conclusion, the tolerance factor can be used as a core geometric indicator for screening stable lead-free double perovskites, and the octahedral factor is an important auxiliary criterion. Therefore, the tolerance factor serves as a primary geometric descriptor for screening stable lead-free perovskites, with the octahedral factor as a key auxiliary parameter.

The geometric window only satisfies topological constraints and cannot reflect thermodynamic stability. By combining SHAP with crystal field theory analysis, the electronegativity at the B site dominates the orbital effect that regulates the formation energy, with geometric matching serving as a secondary factor. Based on this, a geometric–electronic two-dimensional screening criterion for LFDPs is established.

### 2.3. Model Prediction Results

The architectures used in this study are all widely adopted mainstream algorithms. The core contribution of this section is fair benchmarking under unified data partitioning: we synchronously compare four types of DL models with RF and XGBoost in predicting the formation energies of LFDPs, revealing significant performance differences among the six models.

MLP, deep ensemble learning, PINN, and Transformer models are assessed to predict formation energies of LFDPs. Significant discrepancies exist in the predictive performance of these models. As shown in Table 2, the MLP performs best, achieving a mean absolute error (MAE)/mean squared error (MSE)/root mean squared error (RMSE) of 0.0951/0.0189/0.1376 and R^2^ of 0.9147—indicating minimal error and the best fit to the ground truth, capturing nonlinear mappings between electronic descriptors and formation energy. The deep ensemble performs second best (MAE 0.1425, MSE 0.0387, RMSE 0.1966, R^2^ 0.8107), still reliable but worse than MLP. PINN performance declines due to biases from physical constraints (MAE 0.1571, MSE 0.0598, RMSE 0.2445, R^2^ 0.7741). Transformer performs worst on this dataset (MAE 0.1787, MSE 0.0695, RMSE 0.2636, R^2^ 0.7373), with the highest errors and weakest fit. Li [48] trained RF using 5276 groups of ABO_3_ single perovskite, resulting in an MAE of 0.3731 and R^2^ of 0.7231. In this paper, the MLP model was constructed using only 1027 groups of A_2_BB’X_6_ samples. The average error was 25.5% lower than that of the former, significantly improving the fitting effect. This confirms the excellent nonlinear fitting ability of deep learning, which can effectively capture the formation of LFDPs and predict the complex multi-factor coupling effects. It has outstanding application value. For benchmarking, we also constructed classical machine learning models—Random Forest (RF) and XGBoost—based on the same dataset of 1027 sample table entries. The optimal RF achieved a mean absolute error (MAE) of 0.1148 and an R^2^ score of 0.8129 on the test set, while XGBoost obtained an MAE of 0.1179 and an R^2^ of 0.7628. Compared to the multilayer perceptron (MLP), these two shallow models demonstrated significantly lower prediction accuracy, confirming that deep learning’s nonlinear fitting capability is essential for capturing the complex multifactorial interactions involved in predicting the formation energies of A_2_BB’X_6_ LFDPs.

**Figure 4:**
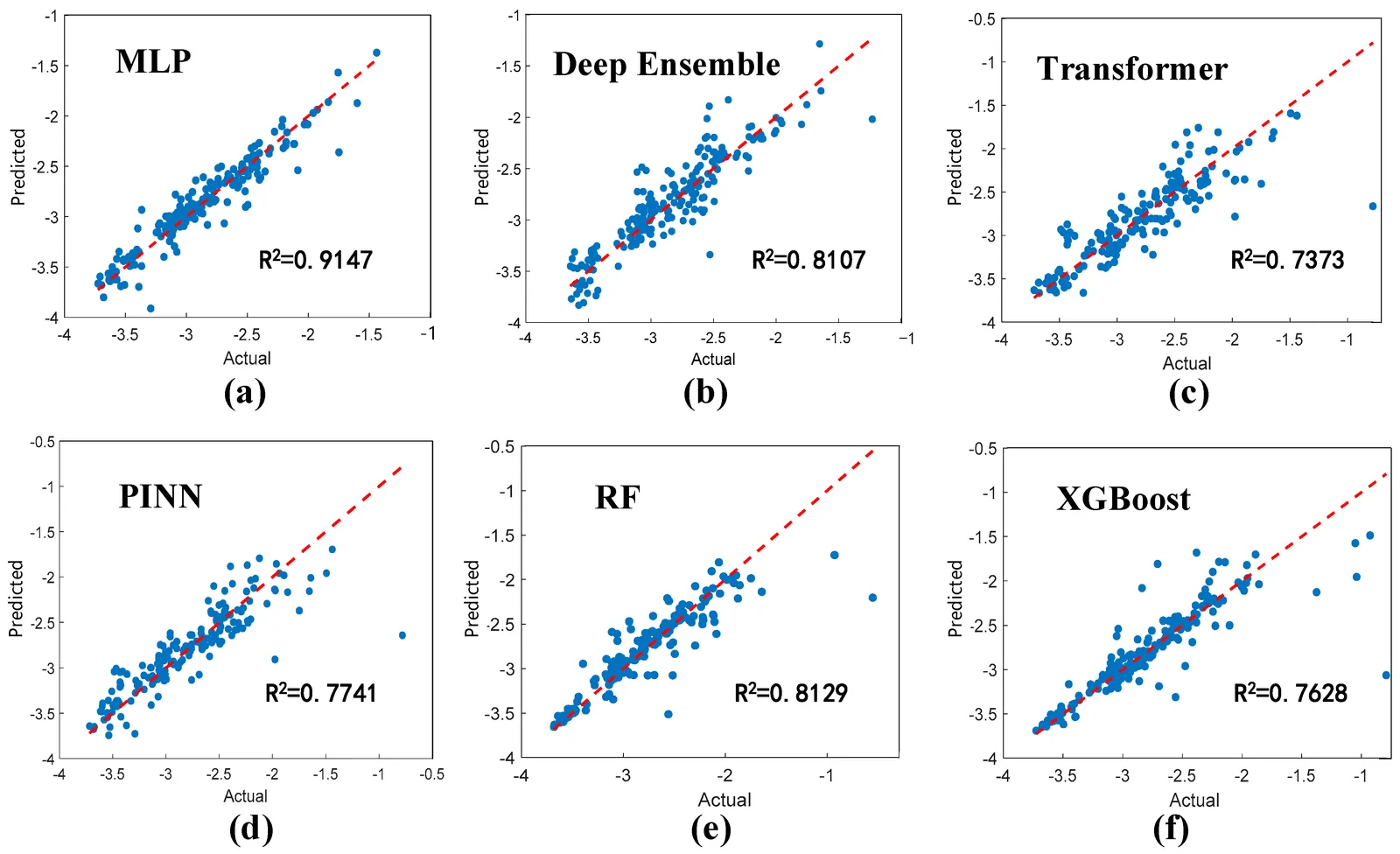
Comparison between actual and predicted values obtained by different models. The blue dots represent the predicted-actual samples, and the red dashed lines denote the ideal 1:1 prediction reference line. (**a**) MLP; (**b**) Deep Ensemble; (**c**) Transformer; (**d**) PINN; (**e**) RF; (**f**) XGBoost.

Figure 4 shows the DFT formation energy as the abscissa and the predicted values of each model as the ordinate. To compare the models and verify the adaptability of the complex network in a 1027-sample small dataset of tabular data, this study employed four DL models (MLP, deep integration, Transformer, PINN) along with two traditional ML models (RF, XGBoost) to predict the formation energy of the double perovskite and compared the predicted results of each model with the DFT true values.

The predicted values for each of the six subplots and the DFT true values all follow the ideal red line (y = x), and all can effectively predict the formation energy. Quantitative R^2^ metrics reveal clear performance hierarchies: the MLP model achieves the highest coefficient of determination (R^2^ = 0.9147), with tightly clustered data points, minimal scatter, tight alignment to the ideal diagonal, and the smallest predictive error. The deep ensemble (R^2^ = 0.8107) and RF (R^2^ = 0.8129) exhibit nearly identical intermediate performance, closely followed by PINN (R^2^ = 0.7741), XGBoost (R^2^ = 0.7628), and Transformer (R^2^ = 0.7373), which ranks as the lowest performing model. Transformer and PINN produce substantially greater point dispersion, with multiple outliers deviating drastically from the ideal fit line, corresponding to a larger prediction bias.

The performance differences among various models stem from the architecture and the dataset. In this study, the small-scale static tabular data have no long sequence features, and the lightweight MLP achieves the best fitting result; the deep integration model has insufficient samples, PINN takes into account the physical constraints and loss to achieve the fitting accuracy, but the Transformer attention mechanism does not apply to tabular features and is prone to overfitting. The performance of RF and XGBoost is inferior to that of MLP. Therefore, the lightweight fully connected MLP is more suitable for this regression task and has the best fitting effect. Moreover, there are fewer samples of transition metals (such as Zr and Ti) in the training set. In this particular system, the prediction bias of complex models is greater, further widening the performance gap among the various models.

In this study, the MLP model was used to predict the formation energies of 20 double perovskites. The results were compared with those calculated using the Perdew–Burke–Ernzerhof (PBE) functional, as shown in Table 3. The results indicate that the overall prediction trend of the model is consistent with the DFT calculation values. The errors for most samples are within the range of ±0.2 eV. Among them, samples such as Ba_2_DySbO_6_ have extremely high prediction accuracy, with an error of only −0.0001 eV. However, oxide systems containing Zr and Ti, such as La_2_CaZrO_6_, have larger deviations, with the maximum error reaching −0.6194 eV. The errors mainly arise from the uneven distribution of some elements in the training data, the insufficient introduction of key physical descriptors such as bond length and coordination field in feature engineering, and the limited ability of the MLP model to capture complex nonlinear interactions such as strong correlation electronic effects and lattice distortion. Additionally, the calculation deviation of the PBE functional for transition metal systems also has a certain impact on model evaluation.

### 2.4. SHAP Interpretability of the Formation Energy Model

Conventional regression metrics (R^2^, MSE, MAE) quantify overall accuracy and generalization but cannot reveal intrinsic links between inputs and formation energy, nor the direction, magnitude, and mechanisms of feature effects [49,50]. Many high-performing nonlinear ensemble models are black boxes with low transparency, limiting mechanistic analysis and precise control. SHAP, based on game-theoretic Shapley values, quantifies feature contributions at both global and local levels, overcoming limitations of traditional methods that cannot separate positive and negative effects [51]. This method defines the model’s predicted value as a linear superposition of all feature contribution values. The core formula is as follows:

(3) $$f(x)=\phi_{0}+\sum_{i=1}^{n}\phi_{i}$$

where *f*(*x*) is the prediction, $\phi_{0}$ is the global average prediction, and $\phi_{i}$ is the SHAP value of feature *i* [52]. SHAP values have clear meaning: positive values increase the predicted target, negative values decrease it, and magnitudes reflect effect strength. Compared with Local Interpretable Model-Agnostic Explanations (LIMEs) and permutation importance, SHAP offers stronger stability and completeness, capturing nonlinearities and interactions, and suits regression analyses [53]. SHAP is adopted on the optimal performing MLP to dissect underlying mechanisms and screen core influencing factors, which delivers mechanistic validation for the scientific rationality of the model.

**Figure 5:**
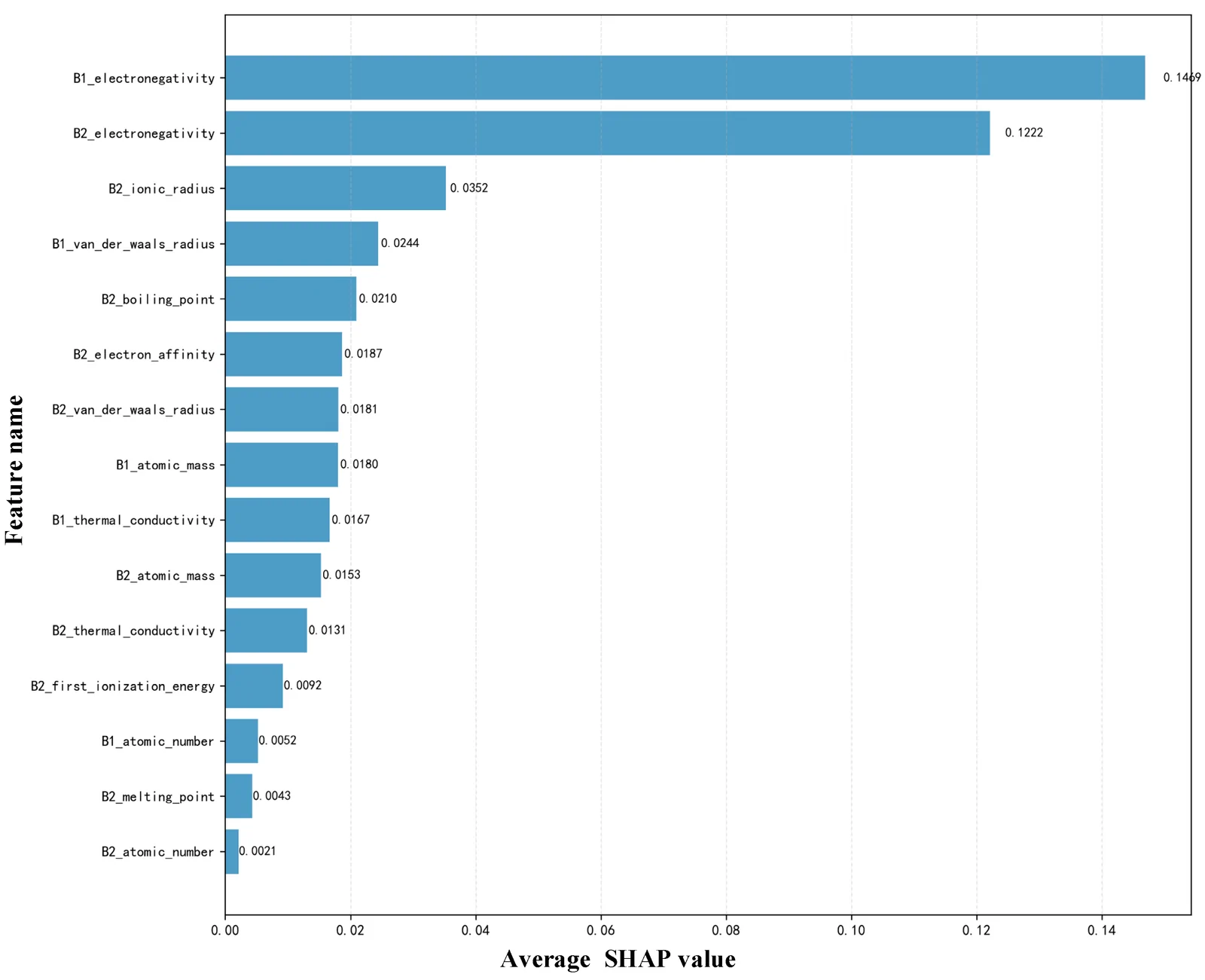
A diagram illustrating the Shapley average value used to form the predictive model.

From the mean absolute SHAP contributions in Figure 5, B1 and B2 electronegativities contribute 0.1469 and 0.1222, respectively, dominating formation energy as core electronic features that govern B-site charge transfer and bond strength. Secondary geometric factors include the B2 ionic radius and B1 van der Waals radius (0.0352 and 0.0244). Boiling point, atomic mass, and thermal conductivity contribute weakly; atomic number and melting point are nearly negligible. Thus, electronic attributes exert a much stronger influence on formation energy than geometric sizes, thermophysical, or basic atomic parameters.

Although the electronegativity at the B site is an important electronic descriptor, its contribution to perovskite stability should be analyzed comprehensively by considering both ionic size and crystal field effects. Specifically, the higher the electronegativity of the B1/B2 cation, the smaller the difference in electronegativity between it and the anions (O/F/Cl), leading to the synchronous enhancement in metal d–anion p orbital hybridization and B-X bond covalency; the increase in covalent bond energy can stabilize the lattice and reduce the formation energy. At the same time, higher electronegativity corresponds to a smaller ionic radius, shortening the B-X bond length and increasing the octahedral splitting energy (Δo). Conversely, low electronegativity B-site ions form ionic chemical bonds, with a smaller Δo, and are prone to a high spin configuration. However, the energy cost generated by this is regulated by the tolerance factor and the size of the A-site ion, and will not necessarily increase the formation energy.

**Figure 6:**
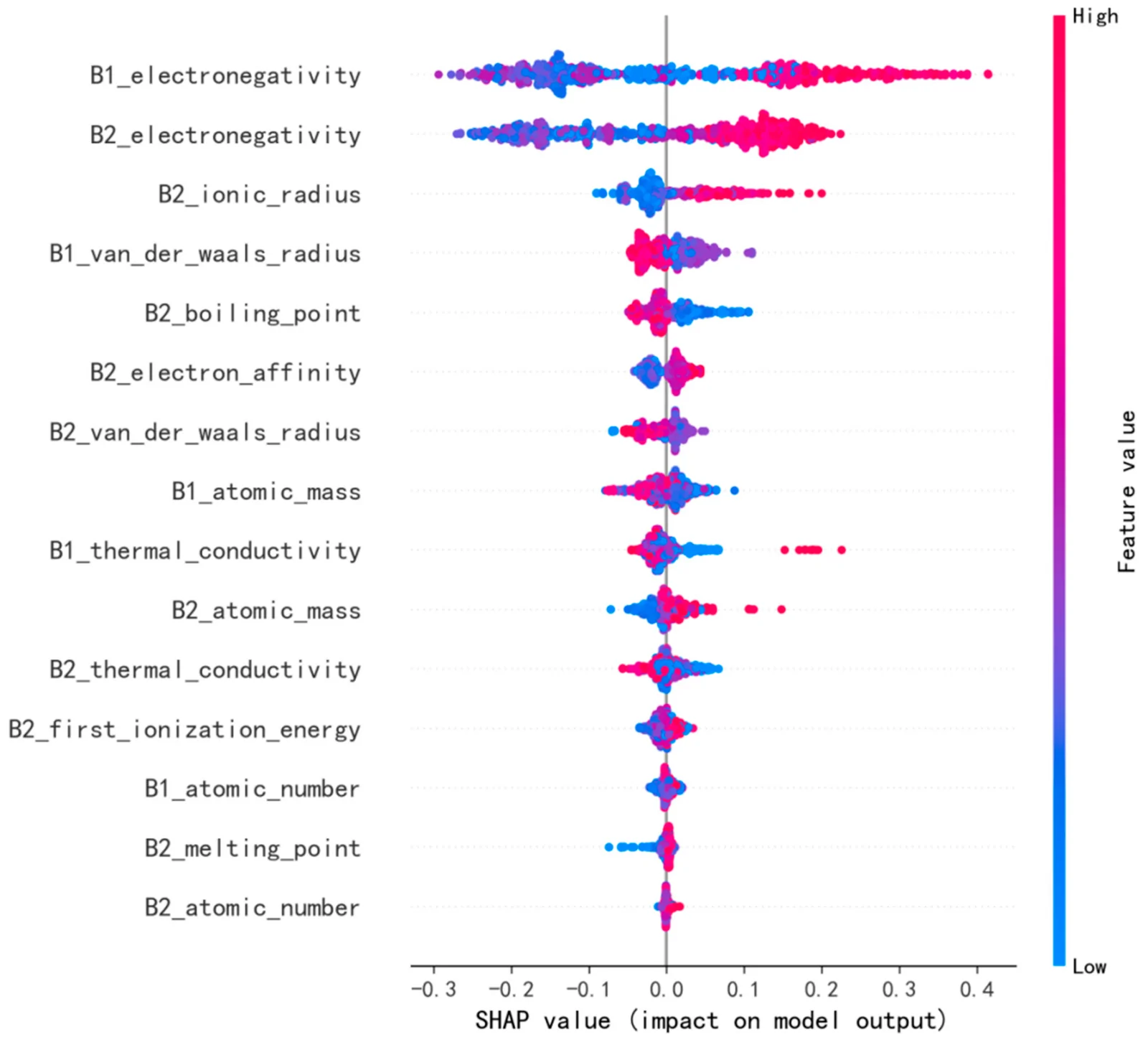
SHAP influence distribution map of formation energy prediction model for LFDPs.

Figure 6 uses SHAP summary plots to quantify the weights of atomic descriptors in predicting lead-free double-perovskite formation energy. Features are ranked by descending average SHAP value, with horizontal axes showing predictive effects and point colors denoting feature magnitudes. B1/B2 electronegativity dominates and favors stability, followed by lattice size descriptors; atomic mass and ionization energy barely affect predictions. Bonding features govern stability far more than geometric parameters, and high values of most features hinder stable perovskite formation.

The trend shown in Figure 6 reflects the chemical rules of the double perovskites. The electronegativities of both B1 and B2 increase simultaneously, resulting in a positive synergistic electron effect. When both B-site cations have strong electron-withdrawing abilities, the crystal field splitting energy (CFSE) of the entire octahedral sub-lattice is uniformly enhanced, and the B1-X and B2-X frameworks form a balanced charge distribution. In contrast, the geometric radius parameters play a secondary restrictive role in regulating the octahedral cage structure. Even if the crystal structure is within the optimal geometric tolerance factor and octahedral factor range, if the electronegativity at the B site is insufficient, it will still lead to higher formation energy and an unstable crystal phase, indicating that electronic orbital interaction is an important factor determining thermodynamic stability.

**Figure 7:**
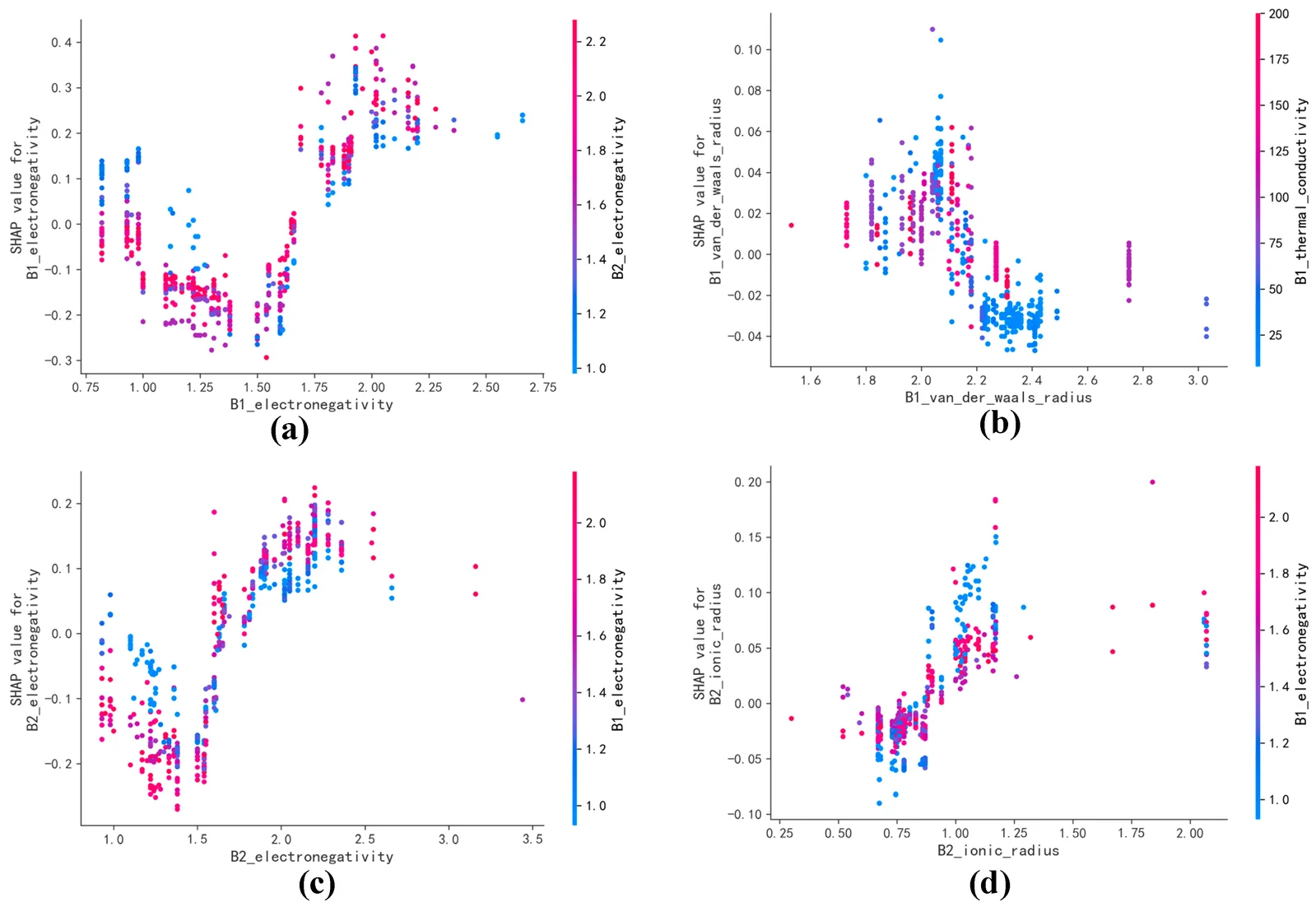
SHAP dependence plots of key features. (**a**) SHAP dependence plot of B1 electronegativity for formation energy prediction. (**b**) SHAP dependence plot of B1 van der Waals radius for formation energy prediction. (**c**) SHAP dependence plot of B2 electronegativity for formation energy prediction. (**d**) SHAP dependence plot of B2 ionic radius for formation energy prediction.

Figure 7 presents partial dependence plots for four key parameters, B1 electronegativity (Figure 7a), B1 van der Waals radius (Figure 7b), B2 electronegativity (Figure 7c), and B2 ionic radius (Figure 7d), revealing nonlinear effects and interactions. For B1 electronegativity, values below 1.6 yield mostly negative contributions; above 1.6, contributions become strongly positive, and higher B2 electronegativity at the same B1 value further increases SHAP. For B2 electronegativity, 1.0~1.6 yields strong negative effects; above 1.8, positive contributions rise steadily, implying that low B2 electronegativity has a broader negative range. Together, they confirm a positive synergistic effect between B1 and B2 electronegativities. For the B1 van der Waals radius, 1.6~2.1 favors improved performance; above 2.2, contributions turn negative rapidly, with high-thermal-conductivity elements forming auxiliary synergy only within moderate size ranges. For the B2 ionic radius, values <0.75 mostly contribute negatively; 0.8~1.5 yields peak positive effects, with slight decline beyond but remaining positive; at the same B2 radius, pairing with higher B1 electronegativity gives higher SHAP. Overall, electronegativity is the core positive driver; selecting high-electronegativity metals at both B sites maximizes gains. Atomic size has an optimal window, and high B1 electronegativity amplifies the positive effects of other physicochemical features—guiding element selection and targeted optimization.

## 3. Data and Methods

### 3.1. Dataset Preparation

#### 3.1.1. Data Source

**Figure 8:**
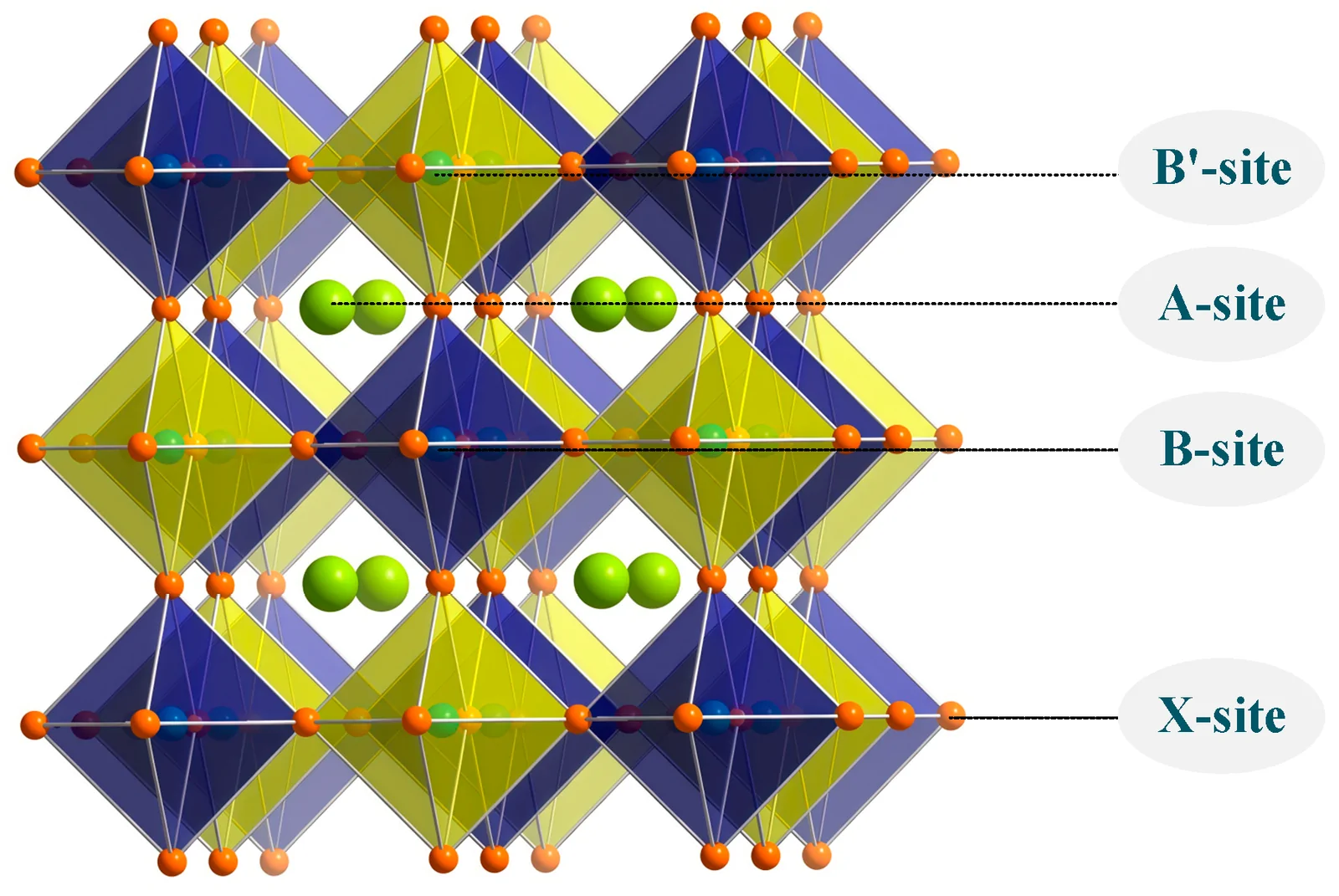
Crystal schematic of A_2_BB’X_6_ LFDPs. Cubic rock-salt-ordered B/B’ sites, cuboctahedral A sites, and X octahedral frameworks. Dataset: A = Ba/Sr/Cs, valence-matched B/B’, X = O/F/Cl for formation energy prediction.

The dataset is compiled from first-principles materials databases and contains 1027 lead-free systems with formation energy as the prediction target. Structures are typical A_2_BB’X_6_ double perovskites (Figure 8). A sites are mainly Ba, Sr, and Cs. B sites are low-valence metals and transition metals; B’ sites are higher-valence transition metals for charge compensation. X sites are mainly O, F, and Cl, ensuring environmental friendliness while covering diverse systems. In terms of the feature dimensions of the dataset, it includes 50 atomic intrinsic features and structural parameters, including the ionic radii and electronegativity of the A, B, B’, and X points, as well as the tolerance factor and octahedral factor, among other structural parameters. The LFDP feature descriptors can be found in Table 1 of Section 2.1. Among them, the formation energy can directly reflect the energy feasibility of material synthesis. In terms of data distribution, it is concentrated within the range of −5 to 0 eV and includes a large number of thermodynamically stable and quasi-stable systems. To ensure the scientific validity of model training and validation, this dataset can effectively support the prediction of the formation energy of LFDPs and the analysis of key features based on DL.

#### 3.1.2. Dataset Splitting

To ensure reliable training and objective evaluation, strict constraints on randomness, distribution consistency, independence, and domain norms were imposed [54]. Following standard practices for materials informatics and DL regression, the dataset was randomly split at an 8:2 ratio via no-replacement sampling, generating 822 training samples and 205 test samples. The training set was used exclusively for parameter updates and optimization of MLP, deep ensemble, PINN, and Transformer models. The test set remained independent, excluded from normalization fitting, training, and hyperparameter tuning to prevent leakage and overestimation. Distribution consistency checks on key indicators (tolerance, octahedral factors) show high overlap and no shifts, reflecting true generalization. This strategy adheres to high-throughput and data-driven modeling norms, enabling fair model comparisons and robust feature analysis.

### 3.2. Deep Learning Algorithms and Model Construction

#### 3.2.1. MLP Algorithm

In predicting the stability of LFDPs, MLPs fit complex nonlinear mappings between descriptors and target energies [55,56]. To achieve rapid and high-throughput assessment of the thermodynamic stability of materials, the formation energy can be used to measure the energy stability of compounds relative to their elemental components. The formation energy calculated by DFT is the output label. The crystal chemical information is transformed into a 50–200-dimensional feature vector as the input layer data *x* = [*x*_1_, *x*_2_, …, *x_d_*]^T^. Then, a fully connected architecture is built. The input layer receives the feature vector; the hidden layer extracts high-order abstract features through linear transformation (Formula (4) [57]) and nonlinear activation (Formula (5)), and the output layer is a single neuron, which is used for regression to predict the formation energy.

(4) $$z^{l}=W^{l}\alpha^{l-1}+b^{l}$$

(5) $$\alpha_{i}^{l}=\max(0,z_{i}^{l})$$

where *l* represents the current network layer number, *z^l^* is the linearly weighted output of the *l*-th layer, *W^l^* is the weight matrix, $\alpha^{l-1}$ is the output activation value of the (*l* − 1)-th layer, *b^l^* is the bias vector, $\alpha_{i}^{l}$ is the final activation output of the *i*-th neuron in the *l*-th layer, and max(0,) is the Rectified Linear Unit (ReLU) activation function. At the same time, regularization methods such as Dropout and BatchNorm are introduced to prevent overfitting. During the training process, the MSE is used as the loss function, and the Adam or RMSprop optimizer is adopted. The network weights are updated through the chain rule of backpropagation (Formula (6)) to minimize the error between the DFT labels and the predicted values. After the training is completed, by inputting the component descriptors of the new lead-free double perovskite, the formation energy can be output in milliseconds, and then stable candidate materials can be screened. Its core advantage lies in the ability to learn complex couplings between atoms through nonlinear activation functions, achieving end-to-end feature learning. However, its limitation is that it does not explicitly encode 3D structure and bond topology. It can be further optimized by combining ensemble learning or transfer learning. Overall, MLP takes descriptors as input, uses a fully connected network as the core, and outputs an energy regression. It is a key algorithm for data-driven discovery of lead-free double-perovskite materials [58].

(6) $$W_{t+1}=W_{t}-\eta \cdot {\hat{m}}_{t}$$

where *W_t_* represents the current weight parameters of the network during the *t*-th iteration of training; *W_t_*_+1_ represents the new weight parameters for the (*t* + 1)-th iteration step; *s* is the learning rate; and ${\hat{m}}_{t}$ is the estimated gradient value obtained from the *t*-th step and backpropagation.

#### 3.2.2. PINN Algorithm

The Physical Information Neural Network (PINN) embeds the physical priors and constraints related to the thermodynamic stability of materials as regularization terms into the loss function of the neural network, achieving the joint driving of data-driven and physical laws [59,60]. It takes descriptors such as material components, structural parameters, octahedral distortion, tolerance factors, etc., as inputs, forms the output target, and constructs a composite total loss function *L_total_* that simultaneously contains the data fitting loss *L_data_* and the physical constraint loss *L_phys_*. PINNs generally weight and sum the data fitting residual and physical residual, with λ serving as the trade-off coefficient:

(7) $$L_{total}=L_{data}+\lambda L_{phys}$$

(8) $$L_{data}=\frac{1}{N}{\sum_{i=1}^{N}\left({\hat{E}}_{f}(x_{i})-E_{f}^{DFT}(x_{i})\right)}^{2}+\frac{1}{N}{\sum_{i=1}^{N}\left({\hat{E}}_{hull}(x_{i})-E_{hull}^{DFT}(x_{i})\right)}^{2}$$

(9) $$L_{phys}=L_{mono}+L_{conserv}+L_{stab}$$

where *L_data_* is the mean square error between the DFT labels and the network predictions [61]. *L_phys_* is achieved through automatic differentiation, and it enforces the satisfaction of the formation energy trend, monotonicity constraint *L_mono_*, decomposition energy conservation constraint *L_conserv_*, and thermodynamic stability criterion constraint *L_stab_* [62].

During the training process, not only are the energy data calculated by the DFT fitted, but also the differential constraints of the input descriptors on the energy surface are satisfied to ensure thermodynamic consistency through the network. Compared with traditional purely data-driven models, PINN can effectively constrain the network output to conform to the material thermodynamic logic, avoiding the occurrence of non-physical energy prediction results. Even in a small-sample DFT dataset, it still has stronger generalization ability and prediction reliability, thereby accurately and efficiently completing the formation energy prediction of lead-free double perovskites, providing a reliable basis for high-throughput screening of thermodynamically stable lead-free perovskite materials.

#### 3.2.3. Deep Ensemble Learning

Deep ensembles train multiple diverse neural networks in parallel and fuse them to reduce overfitting risk and predictive variance, improving accuracy and robustness [63]. Based on DFT-derived labels, the feature set *X* = {*x*_1_, *x*_2_, …, *x_n_*} containing elemental attributes, structural parameters, tolerance indicators and octahedral metrics is constructed. A total of M diverse base models {*f*_1_(*X*), *f*_2_(*X*), …, *f_M_*(*X*)} are generated through three strategies: (i) bagging operation based on bootstrap datasets {*D*_1_, *D*_2_, …, *D_M_*}; (ii) diverse random initialization settings for fixed network architectures; (iii) heterogeneous architectures that capture complementary feature relationships [64]. Each base model is used to fit the nonlinear mapping relationship between the features and the energy indicators:

(10) $$y_{i}^{\left(m\right)}=f_{m}(X_{i};\theta_{m})+\varepsilon_{m}$$

where $y_{i}^{\left(m\right)}$ represents the prediction value of the *m*-th base model for the *s*-th sample, $\theta_{m}$ represents the parameter set of the *m*-th base model, and $\varepsilon_{m}$ represents the model prediction error, which satisfies that the expected value of the error is 0 [65]. During the prediction stage, an integration fusion strategy is adopted to aggregate the outputs of M base models to obtain the final prediction result. Common fusion methods include simple mean fusion (Formula (11) [66]) and weighted fusion (Formula (12) [67]):

(11) $${\hat{y}}_{i}=\frac{1}{M}\sum_{m=1}^{M}y_{i}^{\left(m\right)}$$

(12) $${\hat{y}}_{i}=\sum_{m=1}^{M}w_{m}\cdot y_{i}^{\left(m\right)}$$

where ${\hat{y}}_{i}$ represents the final predicted value of the *i*-th sample, $w_{m}$ represents the weight of the *m*-th base model, and it satisfies $\sum_{m=1}^{M}w_{m}=1$. The weights are usually adaptively allocated based on the performance of the base model on the validation set. At the same time, to quantify the prediction uncertainty, the standard deviation of the output between models is used as the uncertainty indicator, and the formula is as follows:

(13) $$\sigma_{i}=\sqrt{\frac{1}{M-1}\sum_{m=1}^{M}{\left(y_{i}^{\left(m\right)}-{\hat{y}}_{i}\right)}^{2}}$$

where $\sigma_{i}$ represents the prediction uncertainty of the *i*-th sample. The smaller the value of $\sigma_{i}$, the more reliable the model’s prediction result; conversely, this leads to higher uncertainty. Through this indicator, the stable phase and metastable phase of the lead-free double perovskite can be effectively distinguished.

#### 3.2.4. Transformer Algorithm

Transformers use multi-head self-attention to globally model interatomic interactions for accurate thermodynamic stability prediction [68,69]. The core advantage of the Transformer algorithm lies in its self-attention mechanism, which can adaptively capture the relationship between any two elements in a sequence. In crystal structure modeling, this corresponds to the interactions between any pair of atoms [70]. First, atomic embeddings map discrete elemental attributes into vectors, and positional encodings transform 3D coordinates into learnable features:

(14) $$z_{i}=E_{elem}(i)+E_{pos}(r_{i})$$

where *z_i_* is the final feature vector of the *i*-th atom, *E_elem_*(*i*) is the element embedding vector of the *i*-th atom, and *E_pos_*(*r_i_*) is the positional encoding vector based on the three-dimensional coordinate *r_i_* of the atom [71]. This encoding effectively preserves the spatial positions of atoms within the unit cell, laying the foundation for capturing long-range interactions in subsequent steps. After feature encoding is complete, the atomic feature sequence is fed into a multi-layer Transformer encoder, whose core module is multi-head self-attention. This module dynamically calculates the correlation weights between any two atoms through multiple parallel self-attention heads, adaptively capturing the key physical and chemical information that determines the formation energy—including the long-range electrostatic interaction between the A-site cation and the X-site halide anion, the coordination environment and ordered arrangement characteristics of the B1/B2-site metal ions, the bond strength and spatial configuration of the X-site anion skeleton, etc. The calculation process of a single self-attention head is as follows:

(15) $$Attention(Q,K,V)=softmax(\frac{QK^{T}}{\sqrt{d_{k}}})V$$

In this formula, *QK^T^* computes query-key similarity. Dividing by $\sqrt{d_{k}}$ limits logit variance at high dimensions; softmax generates attention weights to weight *V*. *Q*, *K*, and *V* derive from linear projections of atomic features *z_i_*, and *d_k_* is their dimension, whose scaling avoids softmax gradient vanishing. The multi-head self-attention module integrates the outputs of multiple self-attention heads by concatenation and then integrates them through linear transformation to obtain global correlation features [72]. To avoid the problems of gradient vanishing and feature degradation during the training process of deep networks, the Transformer encoder, after the multi-head self-attention module, connects the feedforward network with residual connections and combines them with layer normalization, then abstracts global structural features from the atomic level to the unit cell level layer by layer [73]. The calculation process of the feedforward network is as follows:

(16) $$FFN(x)=\max(0,xW_{1}+b_{1})W_{2}+b_{2}$$

where *W*_1_ and *W*_2_ are linear weight matrices, and *b*_1_ and *b*_2_ are biases; ReLU introduces nonlinearity to enhance the representation ability, and residual connections preserve the information from the shallow layer. Through multi-layer Transformer encoding, the associated features are extracted; global pooling integrates the atomic features into a crystal cell vector; and finally, through the fully connected layer, a regression output is formed to generate the energy.

### 3.3. Model Evaluation

For regression tasks, MSE, RMSE, MAE, and R^2^ are adopted to assess model prediction accuracy and reliability [74]. To fully quantify the model’s fitting performance and predictive stability, four core evaluation metrics are chosen in this work for comprehensive performance quantification.

The MSE is the average of the squared differences between the predicted values and the true values of all samples and is used to measure the overall prediction deviation of the model. This indicator amplifies the errors caused by extreme outliers and highlights the impact of large deviations on the model, providing a quantitative standard for parameter optimization. Its mathematical expression is given by Formula (17) [75]. The RMSE is the square root of the MSE, which standardizes the error dimension relative to the original data. It retains the sensitivity of MSE to outliers and can intuitively reflect the average deviation between the predicted value and the true value, facilitating error analysis and expression, as shown in Formula (18). The MAE is the average of the absolute values of the prediction errors for all samples, which can objectively represent the average prediction deviation of the model. This indicator is resistant to the interference of outliers, and the evaluation results are not easily influenced by extreme values. It is suitable for modeling analysis involving a small number of abnormal samples and can truly reflect the regular prediction effect of the model. Its mathematical expression is given by Formula (19) [76]. The R^2^ is used to evaluate the goodness of fit of the model and to test whether the model is superior to the mean prediction. This indicator is dimensionless and can be used to compare different regression models horizontally. The value range is [0, 1], and the closer the value is to 1, the stronger the explanatory power of the model and the better the fitting effect. Its mathematical expression is given by Formula (20) [77]. It is worth noting that R^2^ will continue to increase as the number of features increases. During the actual evaluation, multiple indicators need to be considered to comprehensively assess the performance of the model.

(17) $$MSE=\frac{1}{n}{\sum_{i=1}^{n}\left(P_{i}-{\hat{P}}_{i}\right)}^{2}$$

(18) $$RMSE=\sqrt{MSE}=\sqrt{\frac{1}{n}{\sum_{i=1}^{n}\left(P_{i}-{\hat{P}}_{i}\right)}^{2}}$$

(19) $$MAE=\frac{1}{n}\sum_{i=1}^{n}|P_{i}-{\hat{P}}_{i}|$$

(20) $$R^{2}=1-\frac{\sum_{i=1}^{n}(P_{i}-{\hat{P}}_{i}{)}^{2}}{\sum_{i=1}^{n}(P_{i}-\bar{P}{)}^{2}}$$

where *n* represents the total sample size, which *P_i_* indicates the true value of the *i*-th sample, ${\hat{P}}_{i}$ represents the predicted value of the *i*-th sample by the model, and $\bar{P}$ is the average of all sample true values.

## 4. Conclusions

This paper focuses on the A_2_BB’X_6_ LFDPs as the research object. The core aim is to establish a reproducible unified multi-model formation energy prediction benchmark without innovating the network structure. Based on a 50-dimensional descriptor dataset containing 1027 sets of DFT data and covering the oxygen/fluorine/chlorine system, using an 8:2 fixed data division, it compares four types of deep learning with RF and XGBoost tree models; combined with SHAP analysis, it quantitatively clarifies the key characteristic laws regulating the formation energy and thermal stability of the materials. Data preprocessing and correlation analysis revealed that the dataset suffered from dimension imbalance, skewed distribution, and multicollinearity. Only factors within specific intervals corresponded to stable crystal structures. Comparative analysis of multiple models showed that MLP performed best (MAE = 0.0951, R^2^ = 0.9147). The error for most samples was ±0.2 eV, with higher deviations in Zr and Ti oxide samples. SHAP analysis indicated that the electronegativity of B-site cations synergistically dominated the formation energy, followed by the lattice size. At the same time, the melting point and atomic mass had a negligible impact. However, this study can only predict the formation energy with a single objective and cannot conduct simultaneous multi-attribute prediction. This work is limited to single-target prediction of formation energy, failing to synchronously predict other critical performance indicators of A_2_BB’X_6_ LFDPs, such as bandgap, decomposition energy, and elastic and phonon properties, which restricts the model’s ability to comprehensively evaluate the material’s structural stability and photovoltaic performance. To overcome this limitation, future work will supplement DFT calculations to enrich the dataset with multi-dimensional stability and optoelectronic descriptors. A multi-task deep learning model will be further developed to achieve simultaneous prediction of multiple material performance parameters, which can effectively improve the model’s comprehensive prediction capability and provide more robust theoretical guidance for the screening and design of high-performance perovskite photovoltaic materials.

## Figures and Tables

**Table 2:** Comparison of the performance results of the regression models.

Algorithm	Evaluation Indicators			
	MAE	MSE	RMSE	R^2^
Deep Ensemble	0.1425	0.0387	0.1966	0.8107
MLP	0.0951	0.0189	0.1376	0.9147
PINN	0.1571	0.0598	0.2445	0.7741
Transformer	0.1787	0.0695	0.2636	0.7373
RF	0.1148	0.0444	0.2108	0.8129
XGBoost	0.1179	0.0616	0.2482	0.7628

**Table 3:** The formation energies of the MLP model in predicting results on the test set.

DPs Order Number	Functional Group	PBE Formation Energy (eV/atom)	Prediction Formation Energy (eV/atom)	Prediction Error (eV/atom)
1	La_2_MgMnO_6_	−3.1847	−3.3989	−0.2142
2	La_2_CrMnO_6_	−3.0783	−3.3495	−0.2712
3	La_2_CoNiO_6_	−2.5702	−2.6847	−0.1145
4	La_2_IrZnO_6_	−2.6512	−2.6269	0.0243
5	La_2_NiZrO_6_	−3.1419	−3.1707	−0.0288
6	La_2_CuSnO_6_	−2.7710	−2.8490	−0.0780
7	Sm_2_CuTiO_6_	−3.0428	−2.9382	0.1045
8	La_2_NaRuO_6_	−2.7896	−2.8085	−0.0189
9	La_2_CaTiO_6_	−3.6790	−3.8012	−0.1222
10	Pr_2_CoRuO_6_	−2.4542	−2.4926	−0.0384
11	La_2_CaZrO_6_	−3.2924	−3.9119	−0.6194
12	Gd_2_TiZnO_6_	−3.3670	−2.9318	0.4352
13	Nd_2_IrNaO_6_	−2.6187	−2.6840	−0.0653
14	Cs_2_ErNaF_6_	−3.6072	−3.5001	0.1071
15	Cs_2_CeNaF_6_	−3.5164	−3.4258	0.0907
16	Rb_2_NaScF_6_	−3.5758	−3.5456	0.0302
17	Rb_2_AgAlF_6_	−3.0536	−2.8969	0.1567
18	Rb_2_NaYF_6_	−3.5704	−3.6245	−0.0541
19	Ba_2_DySbO_6_	−3.0897	−3.0898	−0.0001
20	Ba_2_ErTaO_6_	−3.5980	−3.5551	0.0429

## Data Availability

Data are contained within the article and Supplementary Materials.

## Supplementary Materials

The following supporting information can be downloaded at https://www.mdpi.com/article/10.3390/molecules31183175/s1. Figure S1. Boxplots (a) and density plots (b) of formation-energy-related features.
