# Supplementary material for: Formation Energy Prediction and Feature Analysis of Lead-Free Double Perovskites Based on Deep Learning
Source: Molecules. 2026 Sep 10;31(18):3175. doi: 10.3390/molecules31183175 (PMC13609076; doi:10.3390/molecules31183175)
Supplement: Supplementary file 1 [file molecules-31-03175-s001.zip › molecules-4447966-supplementary.pdf]

Supplementary Data

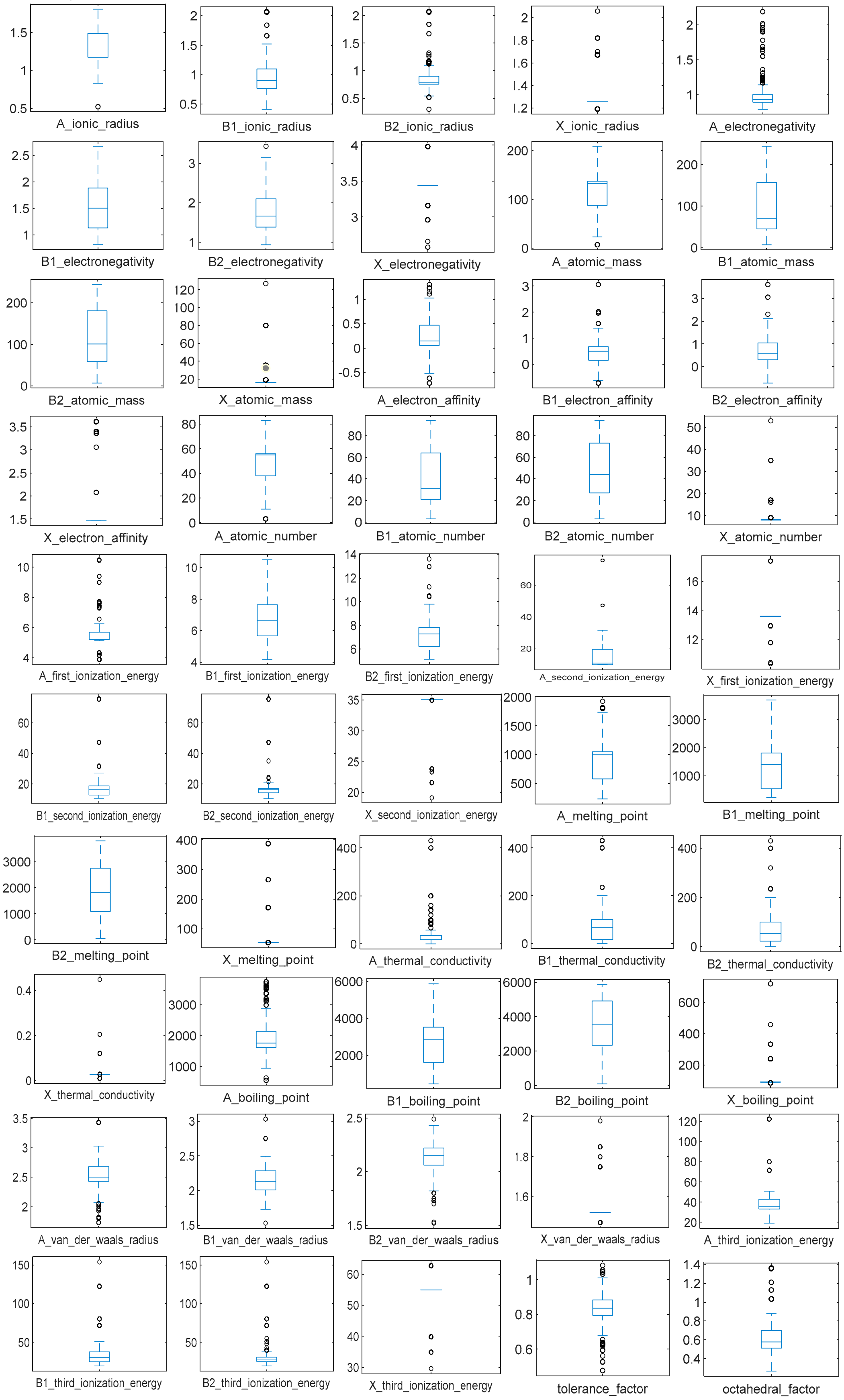

(a)

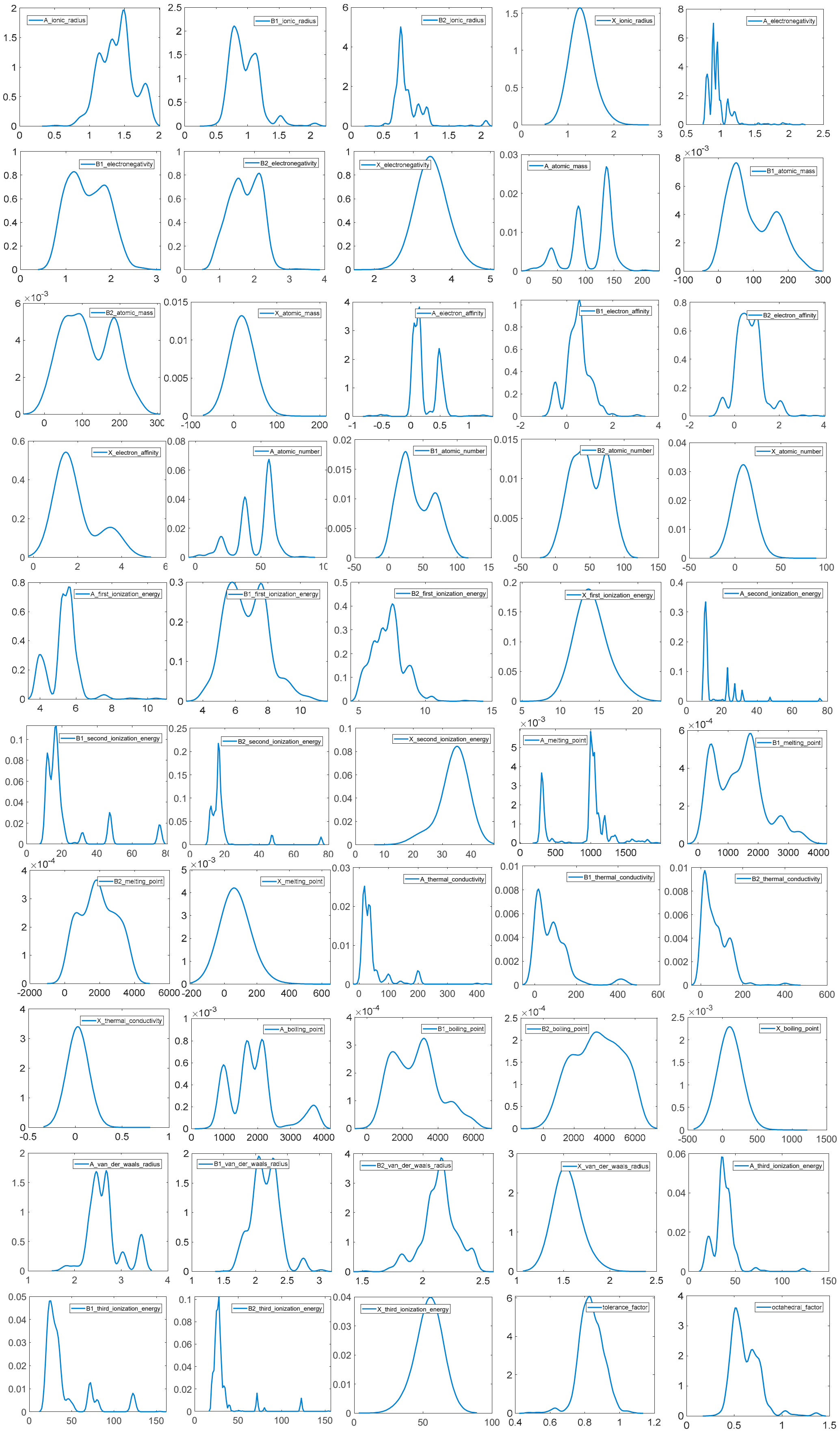

(b)

Figure S1. Boxplots (a) and density plots (b) of formation-energy-related features.
